# Durability Performance of CGF Stone Waste Road Base Materials under Dry–Wet and Freeze–Thaw Cycles

**DOI:** 10.3390/ma17174272

**Published:** 2024-08-29

**Authors:** Zimou Wang, Junjie Yang, Yalei Wu

**Affiliations:** 1 College of Environmental Science and Engineering, Ocean University of China, Qingdao 266100, China; wzm15666820333@126.com (Z.W.); jjyang@ouc.edu.cn (J.Y.); 2 The Key Laboratory of Marine Environment and Ecology of the Ministry of Education, Ocean University of China, Qingdao 266100, China

**Keywords:** stone waste road base material, solid waste-based binder, wet–dry cycles, freeze–thaw cycles, deterioration mechanism

## Abstract

The disposal of stone waste derived from the stone industry is a worldwide problem. The shortage of landfills, as well as transport costs and environmental pollution, pose a crucial problem. Additionally, as a substitute for cement that has high carbon emissions, energy consumption, and pollution, the disposal of stone wastes by utilizing solid waste-based binders as road base materials can achieve the goal of “waste for waste”. However, the mechanical properties and deterioration mechanism of solid waste-based binder solidified stone waste as a road base material under complex environments remains incompletely understood. This paper reveals the durability performance of CGF all-solid waste binder (consisting of calcium carbide residue, ground granulated blast furnace slag, and fly ash) solidified stone waste through the macro and micro properties under dry–wet and freeze–thaw cycling conditions. The results showed that the dry–wet and freeze–thaw cycles have similar patterns of impacts on the CGF and cement stone waste road base materials, i.e., the stress–strain curves and damage forms were similar in exhibiting the strain-softening type, and the unconfined compressive strengths all decreased with the number of cycles and then tended to stabilize. However, the influence of dry–wet and freeze–thaw cycles on the deterioration degree was significantly different; CGF showed excellent resistance to dry–wet cycles, whereas cement was superior in freeze–thaw resistance. The deterioration grade of CGF and cement ranged from 36.15 to 47.72% and 39.38 to 47.64%, respectively, after 12 dry–wet cycles, whereas it ranged from 57.91 to 64.48% and 36.61 to 40.00% after 12 freeze–thaw cycles, respectively. The combined use of MIP and SEM confirmed that the deterioration was due to the increase in the porosity and cracks induced by dry–wet and freeze–thaw cycles, which in turn enhanced the deterioration phenomenon. This can be ascribed to the fact that small pores occupy the largest proportion and contribute to the deterioration process, and the deterioration caused by dry–wet cycles is associated with the formation of large pores through the connection of small pores, while the freeze–thaw damage is due to the increase in medium pores that are more susceptible to water intrusion. The findings provide theoretical instruction and technical support for utilizing solid waste-based binders for solidified stone waste in road base engineering.

## 1. Introduction

**Figure 1:**
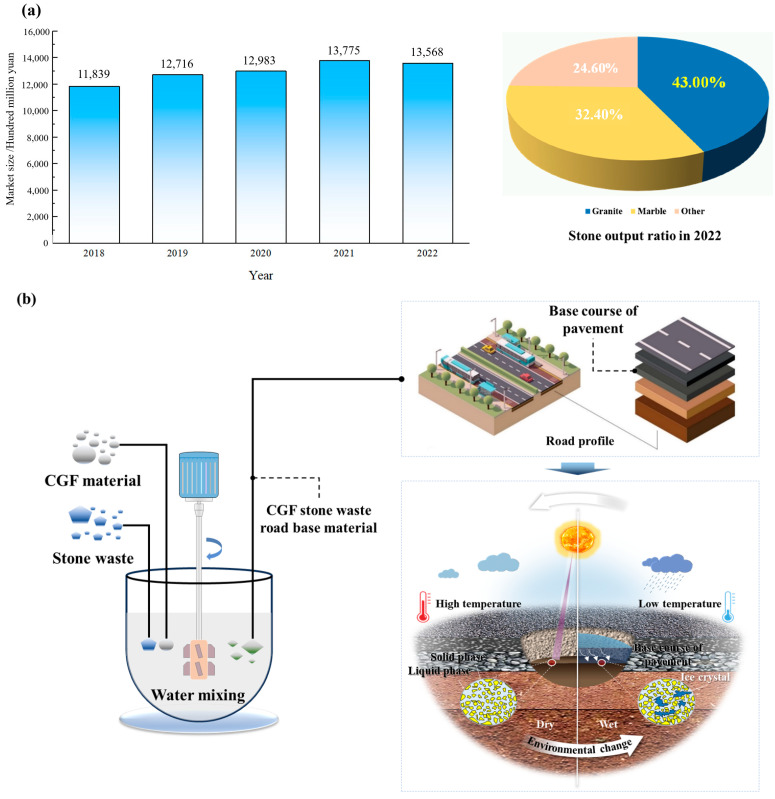
Stone production and road base material durability in China: (**a**) Stone production in China. (**b**) Durability of road base materials.

The disposal of stone waste derived from the stone industry is a worldwide problem [1,2]. China’s stone resources are rich and varied, and according to the statistics of China Mineral Resources Report, in 2021 the mineral reserves of veneer granite were about 16.95 × 10^8^ m^3^, and the mineral reserves of veneer marble were about 5.30 × 10^8^ m^3^, which are ranked among the top in the world [3]. The stone industry is a pillar industry in China’s building materials industry, and its main products are granite stone and marble stone (Figure 1a). China’s granite stone and marble stone production accounts for 39% and 34% of the world, respectively, ranking the highest in the world [4]. The size of China’s specification stone market has been gradually increasing, from CNY 118.39 billion in 2018 to CNY 135.68 billion in 2022, with a compound annual growth rate of 3.5% [5]. With the acceleration of urbanization and the continuous improvement of people’s requirements for the quality of architectural decoration, the market demand for stone products is on a continuous growth trend. The process of finishing slabs involves multiple stages, including ore mining, barren material stockpiling, slab processing, and final slab manufacturing, and stone waste is an inevitable product of stone production. The ratio of stone waste to finished product is about 3:7 [6], and with the increasing amount of stone usage, the amount of stone waste disposal piles is also increasing.

Stone waste is the by-product generated during the production of manufactured sands and due to its high disposal cost and environmental pollution, it is urgent to look for a value-added utilization and green recycling [7,8]; China produces about 859 × 10^4^ t of stone waste in stone processing each year, accounting for 31% of the global total. The granite stone waste generated in granite mining and stone processing exceeds 1000 × 10^4^ t per year [9]. The mineral composition of stone waste particles belongs to primary minerals, and the content of particles larger than 0.075 mm is less than 50%, which is a fine-grained soil without plasticity, while ultrafine stone waste with a particle size of less than 0.005 mm has a certain degree of plasticity. The stone waste particle size distribution range is large but has poor continuity, mostly due to poorly graded soils; among them, some of the ultrafine stone waste is discharged in the form of simple precipitation waste slurry, and the amount of waste slurry accounts for up to 20% and 30% of the weight of barren materials [8]. Stone waste has poor engineering properties and cannot be utilized directly, thus, is often disposed of in stockpiles [10]. In addition to consuming a large amount of land resources and increasing the operating costs of enterprises, the stockpiling of stone wastes may also cause pollution of the surrounding soil, water, and air, posing a threat to human health, as well as landslides and other safety hazards.

Mixing cement and other cementitious materials with stone wastes to prepare concrete [11,12,13] or geotechnical filling materials [14] is the main way to resource stone wastes. The use of stone waste has been reported to significantly reduce the compressive strength of concrete with negligible effect on tensile strength at cement substitution rates ranging from 8 to 10% [15]. Divakar et al. showed that the compressive strength of concrete increased by about 9–11% using stone waste as a partial replacement for natural sand [16].

Campos et al. [17] used stone waste and silica fume to prepare concrete by replacing part of the cement and the test results showed that the strength of concrete was improved when the mixture of stone waste, silica fume, and water did not exceed 33% of the total mass. Chen et al. [18] prepared stone waste concrete, the compressive strength, stiffness, and modulus of elasticity of UHPMC were positively correlated with the MS substitution rate and negatively correlated with the water–cement ratio. In addition, with the increase in stone powder content, the compressive strength showed a tendency of increasing and then decreasing. The best compressive strength was obtained when the stone powder content was 5%. According to the test results of Shen et al. [19], the workability of MS concrete meets the optimum when the content of stone waste is 20%, and the frost resistance and chloride ion penetration resistance achieve the best effect when the content of stone waste is 7%. Bakis [20] used stone waste as an aggregate to make paving stones, with special maintenance methods to improve strength properties and durability. Chen [21] studied the effect of the content of stone waste in mechanism sand on the strength of concrete pavement, and the test results show that concrete pavement has better strength performance and durability when the content of stone waste in concrete pavement is less than 20%.

The resource and energy consumption, ecological damage, and environmental pollution problems caused by cement production are becoming more and more prominent. For every 1 t of cement clinker produced, 1.2 t of limestone, 0.3 t of clayey raw materials, 0.1 t of other raw materials, 0.145 t of standard coal, and 121.58 kW-h of electricity are consumed. In the whole-process environmental emission inventory, the emissions of CO_2_, NOx, SO_2_, CO, and PM10, in order of magnitude, are about 1277.27 kg, 2.87 kg, 2.32 kg, 1.72 kg, and 5.66 kg [8].

On the other hand, according to the Department of Energy Conservation and Comprehensive Utilization of the Ministry of Industry and Information Technology of China in 2022, the annual generation of industrial solid wastes in China is about 3.3 billion t, and the total accumulated historical landfill has reached 60 billion t, covering an area of more than 2 million km^2^, and is mainly distributed in coastal provinces. Among them, China’s production of calcium carbide residue in 2019 is about 2.6 × 10^7^ t [15]; the production of ground granulated blast furnace slag in 2021 is 1.02 × 10^8^ t, with a capacity utilization rate of 39.18%; and the production of fly ash in 2022 is 4.6 × 10^8^ t, with a comprehensive utilization volume of 4.1 × 10^8^ t, with a comprehensive utilization rate of 87.5% [22]. The above-mentioned calcium carbide residue, ground granulated blast furnace slag, and fly ash stacking process also have ecological problems [23].

In summary, the development of alkali-activated cementitious materials (hereinafter referred to as CGF all-solid waste cementitious materials, abbreviated as CGF) whose components are composed of general industrial solid wastes such as calcium carbide residue (CCR), ground granulated blast furnace slag (GGBS), and fly ash (FA), and replacing cement to deal with stone wastes is of great practical significance in realizing the resourceful use of stone wastes and easing the constraints of resources and environment on the economic and social development [23].

The previous research based on CGF has obtained some findings. Wu et al. [24] used CGF (all-solid-waste binder) to solidify pure clay-particle soil, pure silt-particle soil, and pure sand-particle soil, and the test results showed that the solidifying effect was not lower than that of cement (P.O. 42.5). Qiu et al. [25] added fibers to the CGF-solidified soil to further improve the curing ability of CGF. The optimum water content and maximum dry density of CGF-solidified silt were obtained through percussion tests by Liu et al. [26].

Mixing stone waste with industrial solid waste for road paving is currently an effective way of resource utilization. The durability study of road structures [27,28,29] is a long-standing topic in the field of engineering, especially the road base layer to undertake the role of road load transfer, and under the influence of the surrounding environment, its erosion and damage processes involve complex physicochemical processes. The northern region is affected by the natural environment, which is prone to cause engineering problems such as the freezing, thawing, and swelling of salt in road projects. In addition, the accompanying other chemical and biological effects can lead to accidents such as foundation collapse, pavement heaving, and cracking (Figure 1b). Therefore, a large number of scholars have addressed such problems [30,31].

Studies have shown that dry–wet cycles and freeze–thaw cycles have a great influence on the physical and mechanical properties of numerous geotechnical bodies, such as modified soils, roadbed fillers, solid waste-based cementitious materials, etc. The existing studies on the durability of stone waste roadbeds and road base materials are relatively few, and they are mainly focused on the macroscopic strength changes under the influence of dry–wet cycles, freeze–thaw cycles, and the lack of research on the role of microscopic mechanisms. In addition, more and more types of cementitious materials used for cementing stone waste also affect the strength and durability properties of stone waste road base materials, so it is necessary to further study the durability performance of CGF stone waste road base materials and reveal its mechanism of action through microscopic tests.

## 2. Materials and Methods

### 2.1. Materials

**Figure 2:**
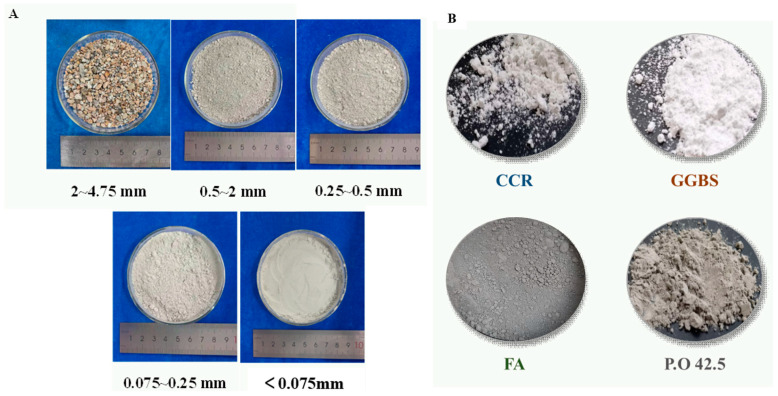
Apparent state of stone waste and cementitious materials. (**A**) Apparent condition of stone waste. (**B**) Figure of cementitious material.

The raw material of stone waste was obtained from Wulian County, Shandong Province, China. In order to avoid the influence of sample differences on the test results as much as possible, the quadratic method was used to obtain representative soil samples randomly. The soil sample retrieved from the site was divided into four equal parts, taken as two diagonal two merged, and mixed into two equal parts in accordance with the same method, and then the two parts were further divided until the test demanded for soil samples. After sampling, the original soil samples were taken for particle size classification and sieved through a sieve to obtain different particle size stone waste (Figure 2A).

**Figure 3:**
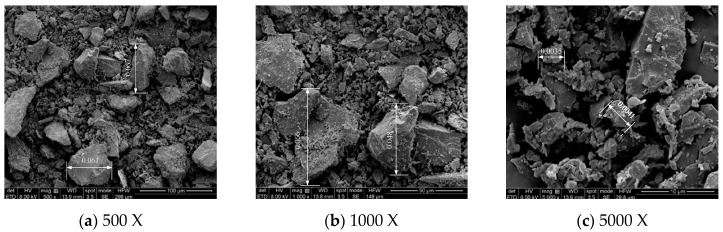
SEM images of stone waste (the size unit marked on the particle in the figure: mm). (**a**) SEM images of stone waste (500 X). (**b**) SEM images of stone waste (1000 X). (**c**) SEM images of stone waste (5000 X).

The micro-morphological characteristics of the Wulian stone waste particles were observed by SEM with different magnifications, and the results are shown in Figure 3. After magnification to 500 times (Figure 3a), the larger particles in the SEM image have been smaller than 0.075 mm in size and are in fine stone dust (0.005~0.075 mm). When magnified to 1000 times (Figure 3b), it can be more clearly observed that the particles of fine stone waste are similar to the appearance of stone chips, which are irregular and multi-angled. When magnified to 5000 times (Figure 3c), the larger particles of the stone waste in the figure are ultrafine stone waste, the surface of the particles is also rough, the shape is irregular and angular, and it is found that there are even finer ultrafine stone waste particles attached to the larger particles in the figure. In summary, the surface of the waste is rough, and the shape is irregular and angular.

Through the liquid–plastic limit experiment, it is concluded that stone waste, stone chips, and fine stone waste have no plasticity, and the liquid limit and plastic limit of ultrafine stone waste are 39.5% and 18.9%, respectively, and the plasticity index is 20.6 (Table 1), the other three kinds of ultrafine stone waste belongs to the high liquid limit powdered soil, while the Wulian ultrafine stone powder belongs to the low liquid limit clay. In summary, the Wulian stone waste belongs to poorly graded soil, which is fine-grained soil without plasticity.

**Figure 4:**
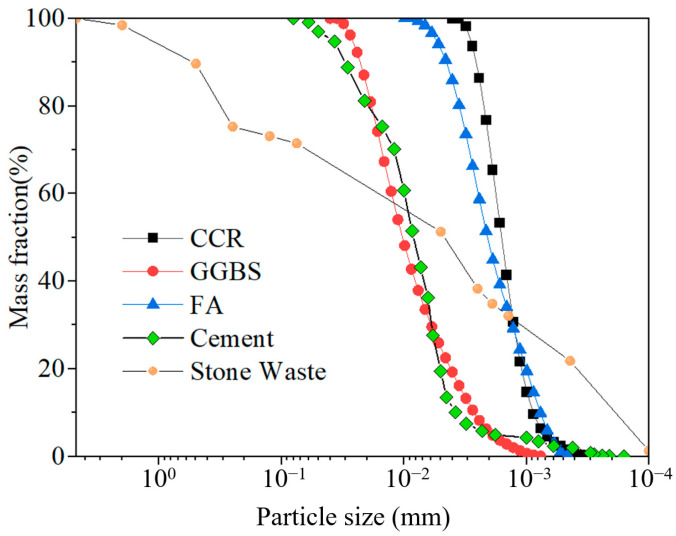
Particle size distribution curves of each component of binder and test soil.

Stone waste in the content of stone chips accounted for 26.2%, and stone waste content accounted for 73.8% (Figure 4). The fine particles smaller than 0.075 mm exceeded 70% of the total weight, indicating that the Wulian stone waste belongs to fine-grained soil; the inhomogeneity coefficient is 85, and the curvature coefficient is 0.42, indicating that the grading of the Wulian stone waste is poor. In order to ensure the accuracy of the test, it is necessary to clean and dry the larger particles of stone waste after sieving in order to avoid the waste and sticky particles attached to the surface of the stone waste particles from affecting the test results.

**Figure 5:**
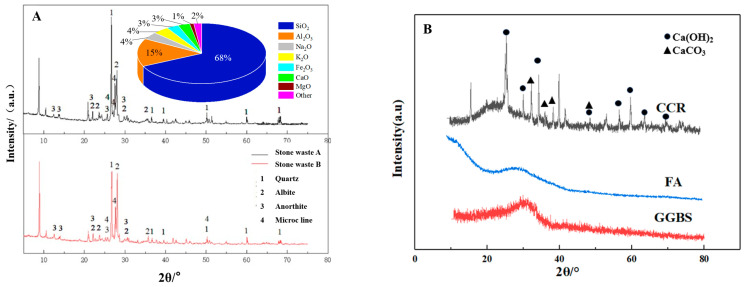
XRD patterns of materials. (**A**) XRD patterns and chemical composition of stone waste. (**B**) XRD patterns of the components of CGF.

In this test, CGF cementitious material was used to compare with P.O 42.5 cement, in which the strength of CGF cementitious material net slurry and solidified pure clayey granular soil, pure powdery granular soil, and pure sandy granular soil is not inferior to cement (P.O 42.5) under the same condition. CGF material consists of alkali exciter and volcanic ash material. Among them, the alkali exciter is in situ calcium carbide residue (CCR), and the volcanic ashy materials are ground granulated blast furnace slag (GGBS) and fly ash (FA). CCR is a by-product of the hydrolysis of calcium carbide to produce ethylene, with a pH value of more than 12.0, which provides an alkaline environment [25]; GGBS is the waste slag produced in the process of iron-smelting, which belongs to the high-reactivity volcanic ashy materials; and FA is the fly ash collected from the baghouses of coal-fired power plants. Collected fly ash, which belongs to low-reactivity volcanic ash material [24]. After the preliminary test, the CGF cementitious material with 4:5:1 of calcium carbide residue (CCR), ground granulated blast furnace slag micro powder (GGBS), and fly ash (FA) was selected for the test. Figure 2B demonstrates the apparent state of stone waste and CGF, Figure 4 shows the gradation curve of the tested materials, and Figure 5 shows the XRD patterns of stone waste and CGF.

### 2.2. Methods

The specimens were prepared as follows:

(1) Raw material treatment: Place the stone waste raw materials in a 105 °C oven drying for more than 24 h to ensure constant quality until its moisture content is not greater than 0.2%.
(2) Specimen preparation: Specimen preparation according to Highway Engineering Inorganic Binding Material Stabilization Material Test procedures (JTG E51-2009) [32]. Specimen molds were used with a diameter of 50 mm and a height of 100 mm.
(3) Standard maintenance: According to the requirements of ‘Test Procedure for Inorganic Binding Material Stabilization in (Highway Engineering (JTG E51-2009)) [32], the standard curing is carried out in a standard curing room with a temperature of 20 °C ± 2 °C and a relative humidity of more than 95%.

The prepared specimens were subjected to mass, height, and diameter measurements, and differences in the mass of parallel specimens shall not exceed 5.0 g. The dry–wet cycle test was carried out using a constant temperature and humidity standard curing box and an electrothermal constant temperature blast drying chamber, and the constant temperature range of the electrothermal constant temperature blast drying chamber was 10~300 °C. The prepared specimens were solidified in the constant temperature and humidity standard curing box for 28 d, with the temperature set at 20 ± 2 °C and humidity ≥95%. The dry–wet cycle of the specimen was divided into two steps of soil sample moisture reduction and soil sample moisture increase, and was repeated continuously according to the number of cycles required for the test, as follows:

(1) Drying stage: The specimen is loaded onto a tray and put into a drying oven with a set temperature of 50 °C for 24 h. After drying, the specimen is taken out and the mass, height, and diameter of the specimen are measured with a balance and vernier calipers. The main purpose of this stage is to remove the moisture of the specimen in preparation for the next stage of hydroponics.
(2) Hydroculture stage: The specimen is immersed in distilled water at a water temperature of 20 °C, and transferred to the standard conservation box (20 °C, 98% relative humidity) for a duration of 24 h. At this time, the specimen is defined as the specimen undergoes the first level of dry–wet cycling test.
(3) Repeat dry–wet cycles: Repeat (1) and (2); when the number of dry–wet cycling levels reaches 1, 3, 6, 9, and 12, select the specimen (parallel samples to take 3) for the unconfined compressive strength test, and the differences in strength shall not exceed 10%. Retain the damaged internal specimen for MIP and SEM microscopic test.

The freeze–thaw cycle test consists of a freezing phase and a hot thaw phase, and the specimen preparation and mass–volume change are carried out in accordance with the steps of the dry–wet cycle test, as follows:

(1) Freezing phase: We wrapped the specimen with cling film and put it into a tray, and then put the tray into a freeze–thaw cycling chamber with a set temperature of −20 °C for freezing. After 24 h, the specimen was removed and prepared for the hot thawing stage.
(2) Hot thawing stage: The specimen was moved to the conservation box with standard conservation, relative humidity 98%, and temperature 20 °C, 24 h after the specimen for the determination of mass, height, and diameter. At this time, the specimen is defined as the specimen undergoes a level of freeze–thaw cycle test.
(3) Repeat freeze–thaw cycle: Repeat (1) and (2), when the number of freeze–thaw cycle levels reaches 1, 3, 6, 9, and 12, select the specimen (parallel samples to take 3) to carry out the unconfined compressive strength test, and the differences in strength shall not exceed 10%. Retain the damaged internal specimen, to be reserved for the MIP and SEM microscopic test.

**Figure 6:**
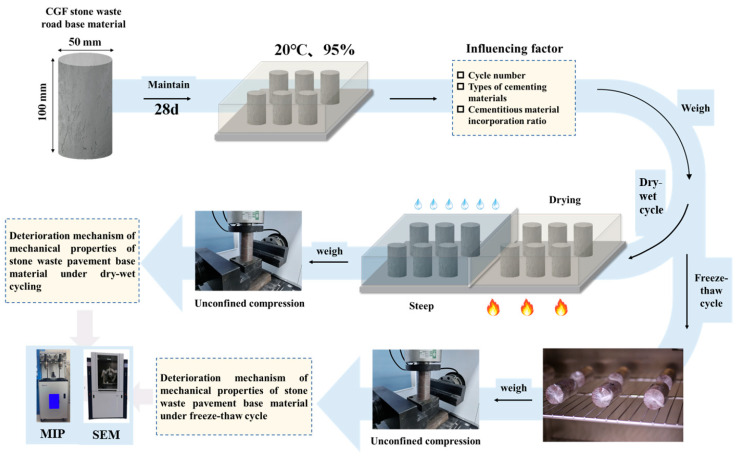
Test process.

In this chapter, the stone waste road base material is set up with two kinds of cementitious materials, CGF and P.O 42.5 cement, and four kinds of mixing ratios of 5%, 10%, 15%, and 20% to study the law of the influence of the dry–wet resistance and freeze–thaw resistance of the stone waste road base material, and a total of eighty groups of specimens are made, three pieces for each group (Table 2), and the test flow is shown in Figure 6.

The unconfined compressive strength test (UCST) was conducted after curing for 7 d and 28 d, which was strain-controlled, and the rate of vertical displacement was fixed at 1%/min. The tests were implemented in accordance with the Standard for Geotechnical Test Methods (GB/T50123-2019) [33]. Dry the raw stone waste in an oven at 105 °C for 24 h, and then mix it with the binder according to the ratio to form a mixture of stone waste and binder. Weigh the required amount of water according to the water–binder ratio of 1.0, add the mixture, and stir evenly for 5 min. After adding water, pour the slurry into the test model in three layers, pounding each layer and chiseling the surface layer of slurry after each round of pounding. Put the specimens into the test model and then into the standard curing box (95% relative humidity, 20 °C) and cure for 24 h. Remove the model after 24 h of curing and continue curing until the set curing time. After curing, subject the specimens to unconfined compression strength, and record the stress–strain curve of each specimen at the same time.

Stone waste road base material blocks were obtained during the compressive test and cut into small slices (10 mm × 10 mm × 10 mm) for conducting the SEM tests. The hydration reaction of the waste material was terminated by soaking in anhydrous ethanol for 7 d. The waste material was dried in an electrically heated blower (50 °C) for 24 days. Drying was carried out in an electric blast oven (50 °C) for 24 h, followed by evacuation and gold spraying, and further observation of the pore structure characteristics of the stone waste road base material was carried out by scanning electron microscopy. The stone waste road base material was cut into cubes (15 mm × 15 mm× 15 mm) for conducting the MIP tests. The relationship between the pore size and pressure can be described by the Washburn equation, see Equation (1). Under pressure, mercury enters the large aperture pores first. *γ_Hg_* and *θ* are invariant, and at higher pressures, mercury enters the small aperture pores gradually. Assuming that the pores in the material are cylindrical, when the pressure is increased from P_1_ to P_2_, the corresponding pore diameters are d_1_ and d_2_, and the volume of mercury pressed into the unit mass of the specimen between the two pore diameters is measured, and when the pressure of the measured pore is varied consecutively, the volume of mercury that enters into different pore diameters can be measured, and thus the pore parameter of the material can be obtained.

(1) $$x=\frac{-4\gamma_{Hg}\pm cos\theta }{d}$$

Equation: P is the applied pressure; *γ_Hg_* is the surface tension of mercury, 0.485 N/m; *θ* is the contact angle of mercury, 130° [34]; d is the pore diameter. Table 3 shows the test instrument parameters and test norms.

## 3. Results and Discussion

### 3.1. Changes in Apparent Characteristics

**Figure 7:**
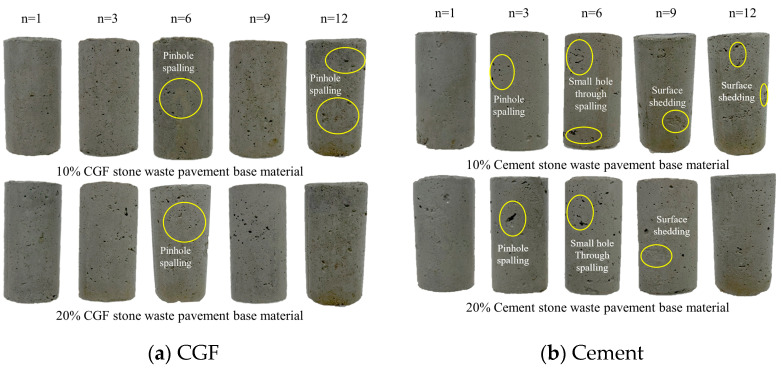
Apparent characteristics of the stone waste road base material under the action of dry–wet cycling: (**a**) CGF stone waste road base material. (**b**) Cement stone waste road base material.

Figure 7a shows the apparent state of CGF stone waste road sub-base materials with 10% and 20% mixing ratios after the corresponding number of dry–wet cycles (n = 1, 3, 6, 9, 12). With the gradual increase in the number of dry–wet cycles, the surface of the specimens was gradually eroded and flaked off, and the above phenomenon was most obvious when the specimens were taken out of the oven for distilled water immersion. At this time, the moisture in the specimen produced a large number of bubbles, accompanied by the phenomenon of specimen peeling. When the mixing ratio was 10%, the surface of the specimen showed small hole peeling, the number was large but the pore diameter was small; when the mixing ratio was 20%, the surface of the specimen also showed small hole peeling phenomenon; compared with the 10% mixing ratio, the area of the peeling increased.

Figure 7b shows the apparent state of the cementitious waste road base material with a 10% and 20% mixing ratio after the corresponding number of dry–wet cycles. With the gradual increase in the number of dry–wet cycles, the surface of the specimen showed the phenomenon of the spalling of small holes and part of the surface peeling off, which occurred at the same time and in the same process as the CGF specimen. When the mixing ratio is 10%, the surface of the cement specimen appears to have small holes through the spalling, and there is a more obvious peeling phenomenon; when the mixing ratio is 20%, the diameter of the small holes on the surface of the cement specimen increases, but the area of the peeling area decreases. From the characteristic analysis of appearance deterioration, CGF specimens did not have a more obvious peeling phenomenon on the surface, and the resistance to dry–wet cycling was better.

**Figure 8:**
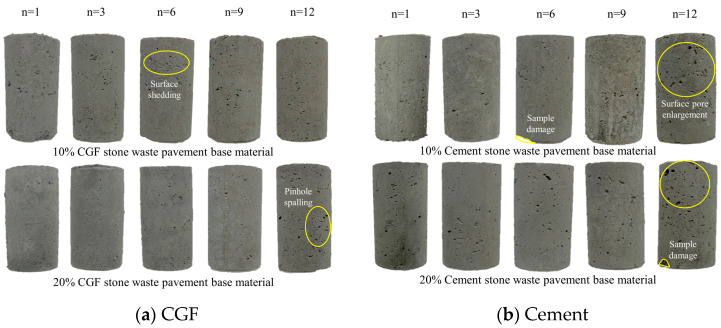
Apparent characteristics of stone waste road base material under freeze–thaw cycle: (**a**) CGF stone waste road base material. (**b**) Cement stone waste road base material.

Figure 8a shows the apparent state of the CGF stone waste road base material after the corresponding number of freeze–thaw cycles (n = 1, 3, 6, 9, 12) at the mixing ratios of 10% and 20%. With the gradual increase in the number of freeze–thaw cycles, the surface of the specimens showed a peeling phenomenon, which was more obvious with the increase in the number of freeze–thaw cycles. Comparing the CGF specimens with a mixing ratio of 10% and 20%, we can find that the specimens with a mixing ratio of 10% have a more serious degree of deterioration in the surface appearance.

Figure 8b shows the apparent state of cementitious waste road base material with 10% and 20% doping ratio after different number of freeze–thaw cycles. With the increase in the number of freeze–thaw cycles, the surface of the specimen also shows small holes peeling off and some breakage in the specimen. The cement specimens treated with freeze–thaw cycles showed a greater degree of apparent deterioration compared to the CGF stone waste specimens.

### 3.2. Mass Loss

Comparing the apparent characteristics of CGF and cementitious waste road base material after different numbers of dry–wet cycles, we can observe that there is a shedding phenomenon on the surface of the specimen, and the area of shedding increases with the increase in the number of cycles. The mass loss rate of different cementitious materials and mixing ratio of stone waste road base material was calculated according to Equation (2):

(2) $$S_{ni}=\frac{Q_{n0}-Q_{ni}}{Q_{n0}}$$

In the equation, *Q_n_*_0_ is the initial mass of the *n*-th specimen, g; *Q_ni_* is the mass of the nth specimen’s *i*-th cycle control sample, g; and *S_ni_* is the *i*-th mass loss of the *n*-th specimen, g.

**Figure 9:**
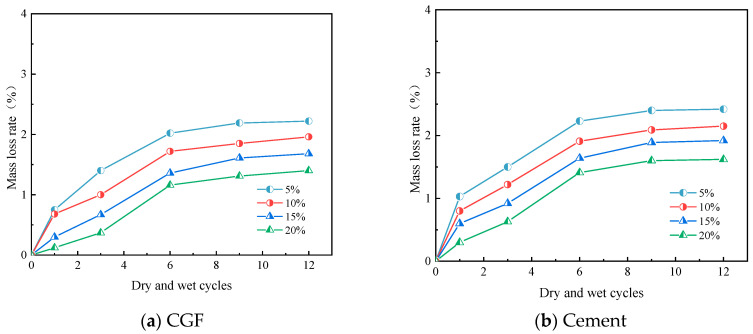
Quality loss rate of stone waste road base material in wet–dry cycle: (**a**) CGF stone waste road base material. (**b**) Cement stone waste road base material.

For the CGF stone waste road base material specimens, the appearance did not change significantly during the dry–wet cycling, and only a small amount of particle shedding occurred on the surface. The mass loss rates of the stone waste road base materials under different CGF mixing ratios are shown in Figure 9a. From Figure 9a, it can be seen that the mass loss rate of the CGF stone waste road base material with the number of dry–wet cycles showed three stages: the first stage of rapid loss stage (1st), when the mass loss rate increased the most, and the mass loss rate of the CGF specimens with the mixing ratios of 5%, 10%, 15%, and 20% reached 1.73%, 1.60%, 1.30%, and 1.05%, respectively; the second slow loss stage, the mass loss rate increased the most, the growth of the mass loss rate slowed down significantly, but there was still a small increase; and the third stage of the stabilization stage, the mass loss in this stage tends to be stable. From the figure, it can be seen that the higher the CGF mixing ratio, the lower the mass loss rate of this specimen, which corresponds to the phenomenon of surface detachment of CGF materials with a low mixing ratio in the apparent deterioration state.

For the cement stone waste road base material, the phenomenon of specimen surface peeling off in the dry–wet process is significantly larger than that of the CGF stone waste road base material. The mass loss rate of stone waste road base material under dry–wet cycling conditions with different cement mixing ratios is shown in Figure 9b, from which it can be seen that the trend in mass loss during the dry–wet cycling of the cement specimens is similar to that of the CGF specimens, which also shows three stages, with the first stage appearing in the 1st dry–wet cycle, the second stage appearing in the 1st~6th dry–wet cycles, and the last stage appearing in the 6th~12th dry–wet cycles. Ultimately, the final mass loss rate was greater than 1% for both types of specimens after the 12th dry–wet cycle.

**Figure 10:**
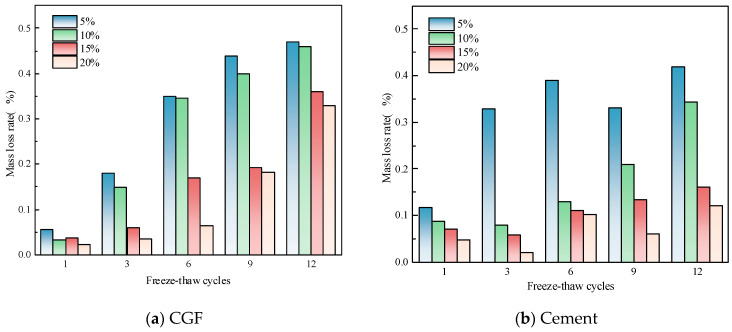
Quality loss rate of stone waste road base material in freeze–thaw cycle: (**a**) CGF stone waste road base material. (**b**) Cement stone waste road base material.

The mass loss rates of the two types of stone waste road base materials after different numbers of freeze–thaw cycles are shown in Figure 10, from which it can be concluded that the mass of the specimens continued to decrease and the mass loss continued as the number of freeze–thaw cycles increased. From Figure 10a, it can be seen that the mass loss rate of the CGF stone waste road base material increased from 0.03% to 0.47% after 1, 3, 6, 9, and 12 cycles at the CGF doping of 5%, and after the CGF doping was increased from 10% to 20%, the mass loss rate under the action of freezing and thawing cycles ranged from 0.00% to 0.46%, 0.01% to 0.31%, 0.06% to 0.31%, and 0.06% to 0.31%, respectively, and 0.06%~0.42%, respectively. From Figure 10b, it can be seen that the mass loss rate of the cement stone waste road base material increased to 0.11%, 0.33%, 0.37%, 0.33%, and 0.42% after 1, 3, 6, 9, and 12 freeze–thaw cycles at a cement dosage of 5%, and the mass loss rate was between 0.05% and approximately 0.42% at a cement dosage of 10% increased to 20%, respectively, 0.02% to 0.16%, 0.06% to 0.34%.

### 3.3. Strength Change

**Figure 11:**
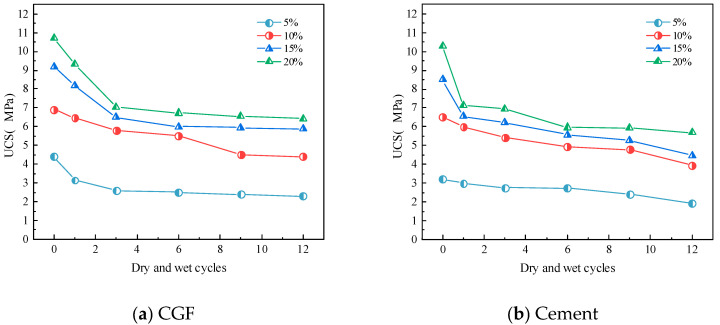
The strength of stone waste road base material varies with the number of dry–wet cycles.: (**a**) CGF stone waste road base material. (**b**) Cement stone waste road base material.

Figure 11 shows, respectively, for the specimens, the unconfined compressive strength with the number of dry–wet cycles in a change curve; with the number of dry–wet cycles increasing, the unconfined compressive strength of the CGF and cement stone waste road base material specimens is gradually reduced, and in the first six dry–wet cycle stage, the rate of change is larger, and with the number of dry–wet cycles continuing to increase, the specimens’ rate of the gradual reduction in unconfined compressive strengt. The main reason is that when the specimen is subjected to evaporation and humidification in the dry–wet cycles, the internal microcracks continue to develop, and the compressive strength decreases. With the increasing number of dry–wet cycles, the structural strength of the specimens increases with the degree of deterioration and finally tends to stabilize.

**Figure 12:**
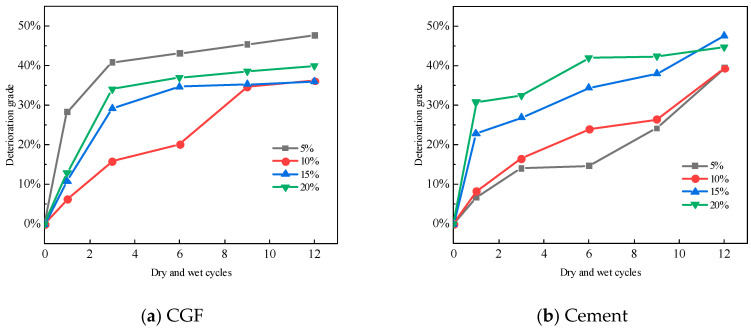
The deterioration degree of stone waste road base material changes with the number of dry–wet cycles: (**a**) CGF stone waste road base material. (**b**) Cement stone waste road base material.

In order to quantify the deterioration characteristics of the unconfined compressive strength of CGF stone waste and cement stone waste road base materials under dry–wet cycling conditions, the deterioration of the strength of the stone waste road base materials was obtained by calculating the strength parameter deterioration (DG) (Figure 12) as shown in Equation (3):

(3) $$DG_{c(q)}=\frac{c{\left(q\right)}_{0}-c{\left(q\right)}_{i}}{c{\left(q\right)}_{0}}\times 100\%$$

In the equation, *c*(*q*)_0_ is the compressive strength of the specimen without dry–wet cycles; *c*(*q*)*_i_* is the compressive strength of the specimen after the different numbers of dry–wet cycles.

**Figure 13:**
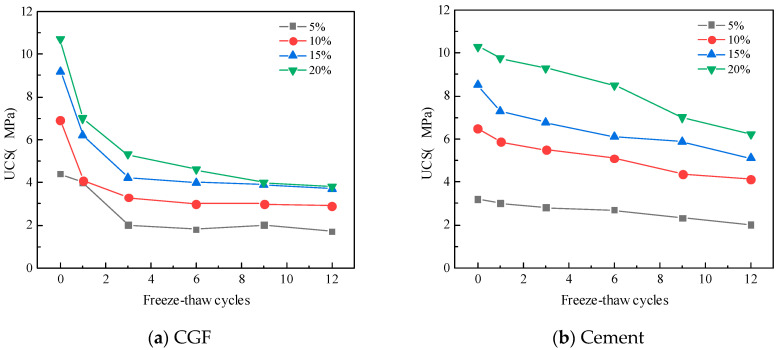
The strength of stone waste road base material varies with the number of freeze–thaw cycles: (**a**) CGF stone waste road base material. (**b**) Cement stone waste road base material.

According to Formula (2), the deterioration of the two types of stone waste road base materials with dry–wet cycling is shown in Figure 13. For the CGF stone waste road base materials, the deterioration of the specimens ranges from 36.15% to 47.72% under the condition of four CGF mixing ratios after 12 dry–wet cycles. For cement stone waste road base materials, after 12 dry–wet cycles, the deterioration of the specimens ranged from 39.38% to 47.64% for the four cement mixing ratios. As can be seen from Figure, both the CGF stone waste road subgrade material and cement stone waste road subgrade material have a large increase in damage deterioration at the beginning of the dry–wet cycles, and the growth rate gradually decreases with the increase in the number of dry–wet cycles. For CGF stone waste road base material, the deterioration degree was 47.7% after 12 cycles with a CGF doping ratio of 5%, and decreased to 36.2% with a mixing ratio of 10%.

After the freeze–thaw cycles, the test results of the unconfined compressive strength of the stone waste road base materials under different cementitious material mixing ratios are shown in Figure 13. As can be seen from Figure 13, the unconfined compressive strength of the two types of stone waste road base materials decreases in a non-linear manner with the increase in the number of freeze–thaw cycles, and the main loss of strength occurs after the first cycle: the average loss of strength of the CGF stone waste road base material after the first freeze–thaw cycle reaches 25.86%, whereas the average loss of strength of the cemented stone waste road base material under the same conditions is 11.65%.

The statistical results show that the average unconfined compressive strength of CGF stone waste road base material with 5% to 20% dosage was reduced to 25.86%, 8.36%, 11.89%, and 9.36% with the increase in the number of freezing and thawing cycles in the order of the average unconfined compressive strength of CGF stone waste road base material with 5% to 20% dosage. The mean unconfined compressive strength of the CGF stone waste road base material was reduced by 11.65%, 7.13%, and 6.72% for 1 to 6 freeze–thaw cycles. In general, the strength of the two types of stone waste road base materials tends to stabilize after six freeze–thaw cycles. The decrease in the strength of the freeze–thaw cycle specimens was caused by the microstructure of the materials. In the freezing process, after the water changes from liquid to solid, the pore water between the particles expands in volume, which produces a tensile effect on the specimen as a whole, and when the expansion force is greater than the tensile strength of the pore skeleton, it will result in the skeleton crushing or displacement, and the connecting characteristics between the particles will change, and at the same time, it will cause the volume of the original pore in the specimen to expand, and even produce a fissure when it is penetrated. During the thawing process, the pore volume of the soil cannot be fully restored, so repeated freezing and thawing destroys the initial structure of the material and generates a new structure, leading to the overall strength decay of the specimen. With the increase in the number of freeze–thaw cycles, the internal structure of the stone waste specimen tends to stabilize, and the macroscopic manifestation is that the strength also tends to stabilize.

**Figure 14:**
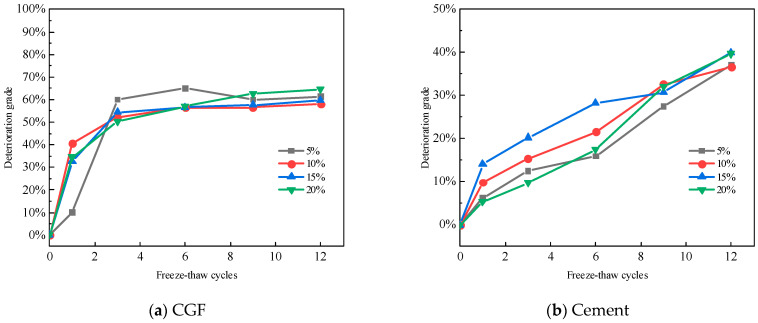
The deterioration degree of the stone waste road base material changes with the number of freeze–thaw cycles. (**a**) CGF stone waste road base material. (**b**) Cement stone waste road base material.

Similar to the calculation of the deterioration degree of dry–wet cycles, the deterioration degree of the specimen after a freeze–thaw cycle is obtained from Equation (2), and the deterioration degree of the two kinds of stone waste road base materials in the freeze–thaw cycles is shown in Figure 14:

It can be seen from Figure 14 that for the CGF stone waste road subgrade materials, the deterioration of the specimens under 4 CGF mixing ratios ranged from 57.91% to 64.48% after the 12 dry–wet cycles. For the cementitious stone waste road subgrade materials, after the 12 freeze–thaw cycles, the deterioration of the specimens ranged from 36.61% to 40.00% for the four cement incorporation ratios. It can be seen that the resistance of the CGF stone waste road base material to the freeze–thaw cycles is weaker than that of the cement stone waste road base material.

### 3.4. Microstructure Analysis and Deterioration Mechanism

(1) Analysis of MIP test

**Figure 15:**
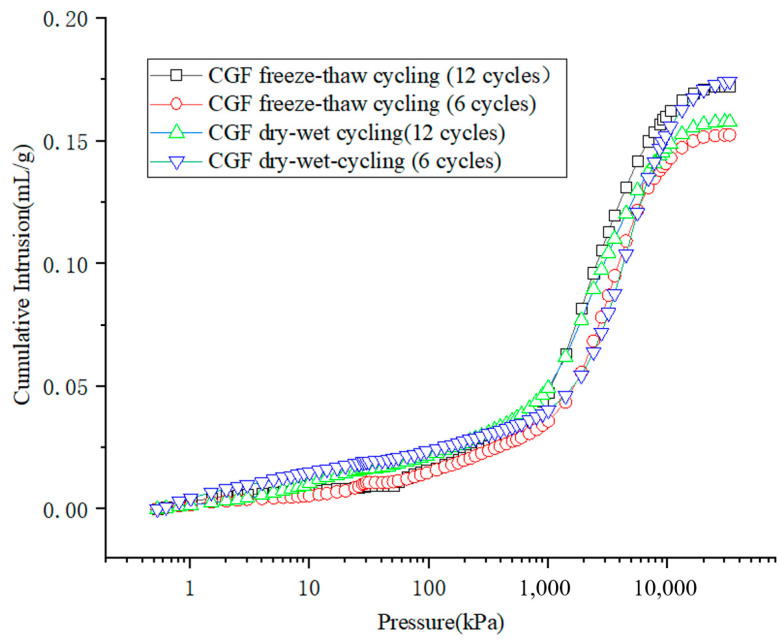
Relationship curves between cumulative mercury intake and pressure.

The cumulative mercury feed versus pressure curve is shown in Figure 15. Since mercury is a non-infiltrating liquid, it can enter the pores of the stone waste road base material under pressure. It can be analyzed that the cumulative mercury feed increases with increasing pressure. When the pressure is small, mercury can enter the pore space of large pore size, and the cumulative amount of mercury feed is small; of course, this is also closely related to the pore structure of the material. When the pressure reached 4.01 kPa, mercury rapidly entered into the pores of the stone waste road base material. However, the pressure required for the rapid entry of mercury into the pores of the cement stone waste road base material is close to 1393.1 kPa. The large slope of the curve characterizes the rapid entry of mercury into the pores at this pressure, i.e., there are more pores in the range of pore size distribution.

The pore size distribution determines the pore structure characteristics [37]. Using the pore classification method, the pores in coal are classified into micropores (<10 nm), small pores (10~ < 100 nm), medium pores (100~1000 nm), and large pores (>1000 nm) [38].

**Figure 16:**
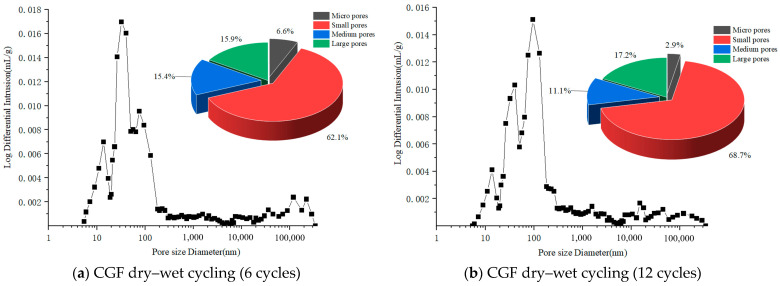

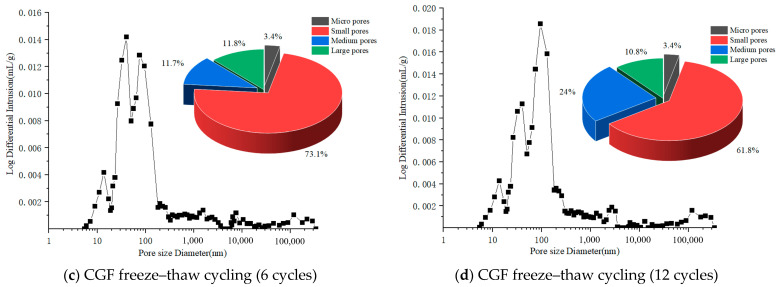
The pore diameter differential distribution curves of the stone waste road base materials.

As shown in Figure 16, the void structure of the stone waste road base material is mostly composed of micropores and small pores. With the increase in the number of wet–dry and freeze–thaw cycles, the void volume gradually increases, with small pores progressing to medium pores. As shown in Figure 16, the pore structure of the stone waste road base material after 12 times of wet–dry cycling was mostly composed of small and large pores. With the increase in the number of wet–dry cycles, the volume of micropores and mesopores gradually decreases, and the volume of small pores and macropores gradually increases (Figure 16a,b). As shown in Figure 16, the pore structure of the stone waste road base material after 12 cycles of dry–wet cycling was mainly composed of small pores and mesopores. With the increase in the number of freeze–thaw cycles, the volume of the small pores gradually decreased, the volume of mesopores increased significantly, and the change in the volume of micropores and macropores was not obvious (Figure 16c,d).

The differential distribution curve of the pore size of the stone waste is shown in Figure 16. From the analysis results, it can be seen that each curve has a main peak, i.e., the pore size corresponding to the main peak is dominant in the pore size of the stone waste. The pore sizes of the main peaks were 40.35 nm (Figure 16a) and 75.65 nm (Figure 16b) for 6 and 12 times of dry–wet cycles, respectively; the pore sizes of the main peaks were 56.77 nm (Figure 16c) and 95.62 nm (Figure 16d) for the freeze–thaw cycles, indicating that the main peaks were gradually shifted to the right with the increase in the number of cycles.

The test results show that at the beginning of the deterioration reaction, the dry–wet cycles and the deterioration reaction caused the deformation of the specimen and the generation of small cracks, and with the increase in the number of cycles, the cracks increased, the hydration products were detached from the surface of the stone wastes with the action of the dry–wet cycles, and the adhesion between the particles was decreased, which also led to the decrease in the strength of the material. The deterioration reaction of the stone waste under the freeze–thaw cycles is more obvious, which leads to a significant increase in the percentage of mesopores.

**Figure 17:**
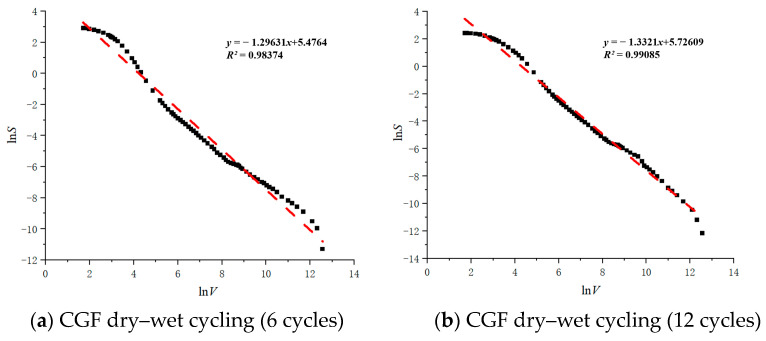

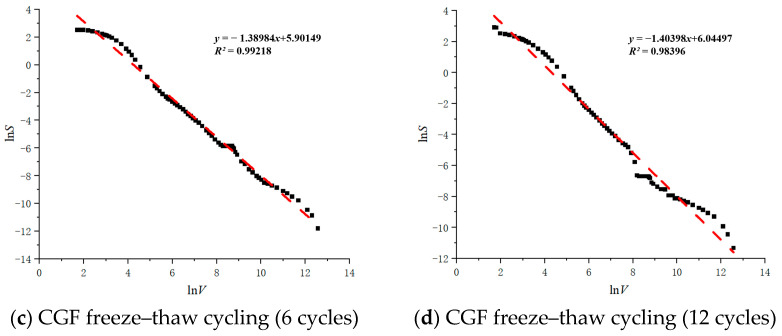
The relationship between *lnV* and *lnS*.

It was shown that the V–S model is suitable for analyzing the relationship between Cumulative Pore Area (*S*) and pore size (*V*) volume applicable to micropore and small pore media. The slope of the fitted curve (*lnV*–*lnS*) increases with the number of dry–wet and freeze–thaw cycles (Figure 17), which suggests that the development of porosity is a function of the number of cycles for the practical application of stone wastes.

(2) Analysis of SEM test

**Figure 18:**
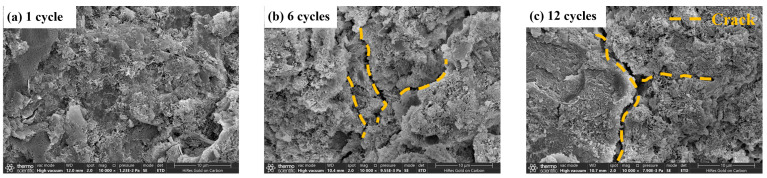
Schematic diagram of microstructure evolution of CGF stone waste road base material sample under dry–wet cycling.

Changes in the microstructure of the material will cause changes in the macro-mechanical properties; in order to study the strength change mechanism of the stone waste road base material under the action of dry–wet cycling, two kinds of stone waste road base material were observed and studied by SEM test. Figure 17 and Figure 18 show the microstructure of the cement stone waste specimen and CGF stone waste specimen after different times of dry–wet cycles; it can be found that after the dry–wet cycles, there are no cracks generated inside the specimen at first, and with the increase in the number of dry–wet cycles, cracks are gradually generated inside the specimen, and the observation of the internal structure of the specimen reveals that the amount of hydration products decreases with the increase in the number of cycles because the hydration products are removed from the stone waste particles by the washing action of the dry–wet cycles, and the hydration products are removed from the stone waste particles by the washing action. This is because the hydration products are detached from the surface of the waste particles by the scouring action of the dry–wet cycles, and the cohesion between the particles is reduced as a result. With the increase in the number of cycles, the number of small cracks inside the specimen becomes larger, the distribution range becomes wider, and eventually, a through pore is formed, and the adhesion between the particles decreases and gradually separates, resulting in a decrease in the overall performance of the specimen, which leads to a significant reduction in the strength of the material.

**Figure 19:**
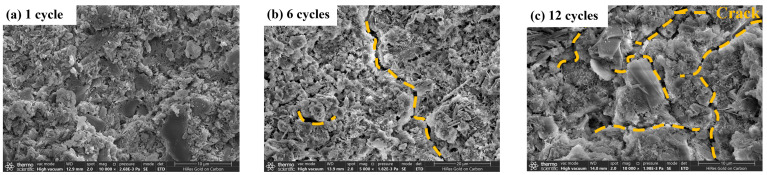
Schematic diagram of microstructure evolution of cement stone waste road base material sample under dry–wet cycling.

Figure 18 and Figure 19 show the development of the internal deterioration of the specimen under different numbers of dry–wet cycles for the stone waste base material. After 1 dry–wet cycle, the CGF stone waste specimen (Figure 18a) and the cement stone waste specimen (Figure 19a) are relatively stable internally, and there is no obvious cracking; after 6 dry–wet cycles, the CGF specimen (Figure 18b) and the cement stone waste specimen (Figure 19b) show cracks internally, and the deterioration of the cement stone waste specimen (Figure 18c) is the same as the CGF stone waste specimen (Figure 19c) after 12 dry–wet cycles. The deterioration of the CGF stone waste specimen (Figure 18c) is similar to that of the cement stone waste specimen (Figure 19c) in that the degree of deterioration increases with the increase in the number of dry–wet cycles, and the degree of cracking in it intensifies.

Comparison with the previous strength results shows that the stone waste specimens received a synergistic effect of moisture and temperature during the dry–wet cycling process, resulting in a rapid increase in strength. Under 3 dry–wet cycles, the strength loss increased considerably; after 12 dry–wet cycles, the magnitude of the strength loss rate of the stone waste specimens slowed down, and the compressive strength of the specimens decreased slowly. In the early stage of the deterioration reaction, the dry–wet cycles caused the specimen’s deformation, resulting in the generation of small cracks, but the hydration reaction occurring inside the specimen coupled with the dry–wet cycle action played a compensatory role, so the magnitude of the strength loss was small in the case of a small number of dry–wet cycles. With the increase in the number of dry–wet cycles, the cracks continued to increase and the degree of cracking intensified, leading to a reduction in compressive strength. After 12 cycles of dry–wet cycling, the strength change in the specimen tends to stabilize, the CGF stone waste road base material has lower strength loss and better stability compared to the cement stone waste road base material, and under alkali excitation, the CGF stone waste road base material has a higher potential for later hydration reaction, which better reduces the loss of strength, i.e., the resistance to dry–wet is better.

**Figure 20:**
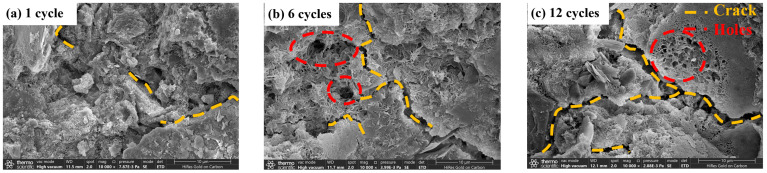
Schematic diagram of microstructure evolution of CGF stone waste road base material sample under freeze–thaw cycling.

**Figure 21:**
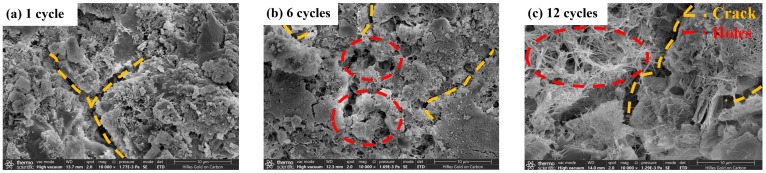
Schematic diagram of microstructure evolution of cement stone waste road base material sample under freeze–thaw cycling.

Figure 20 and Figure 21 show the microstructure of the CGF stone waste specimens and cement stone waste specimens after different freeze–thaw cycles. It can be found that after 1 freeze–thaw cycle (Figure 20a and Figure 21a), the surface of the specimen is relatively flat, and only a small number of cracks are generated; with the increase in the number of cycles, after 6 freeze–thaw cycles (Figure 20b and Figure 21b), a large number of holes and cracks appear on the surface of the specimen, and the macroscopic manifestation of the specimen is the rapid loss of the mass and the compressive strength of the specimen which is decayed; after 12 freeze–thaw cycles (Figure 20c and Figure 21c), the specimen surface has a large number of holes and cracks. After 12 freeze–thaw cycles (Figure 20c and Figure 21c), the free water inside the specimen accelerates the expansion of the microcracks and the change in crack properties due to the expansion stress generated during the freeze–thaw cycle, thus accelerating the deterioration of material properties, and the micro-morphological characteristics show a dense pore structure.

Comparing the results of the previous strength tests, the unconfined compressive strength started to decrease significantly as the number of freeze–thaw cycles increased to 6, and the strength of the specimens stabilized after 12 freeze–thaw cycles. This is because during the standard maintenance process, the specimen produces more cement, which gels and wraps the soil particles and fills the pores between the soil particles, making it more difficult for free water to enter, which significantly improves the soil’s ability to resist freezing and thawing. When the number of freeze–thaw cycles is small, internal cracks begin to appear because the hydration reaction caused by the degree of strength enhancement is greater than the degree of strength deterioration caused by the freeze–thaw cycle, so the overall unconfined compressive strength is a slow increase in the state, i.e., the hydration reaction that occurs within the specimen coupled with the freeze–thaw cycle. With the increasing number of freeze–thaw cycles, the original cracks intensified and more cracks appeared, the saturation degree of the soil body continued to increase, and the freezing and expansion effect intensified, while the hydration reaction ceased, resulting in the beginning of the downward trend in the unconfined compressive strength. On the other hand, with the increase in the number of freeze–thaw cycles, the void volume inside the specimen gradually increased, but the number of mesopores and void structure remained relatively stable. This is because once the bonding between the hydration product crystal particles is broken, sufficient space is provided for water molecules to transform into ice. As a result, the remaining hydration product crystal particles are no longer damaged by the freeze–thaw cycles, allowing the strength of the stone waste road base material to reach a state of dynamic equilibrium.

**Figure 22:**
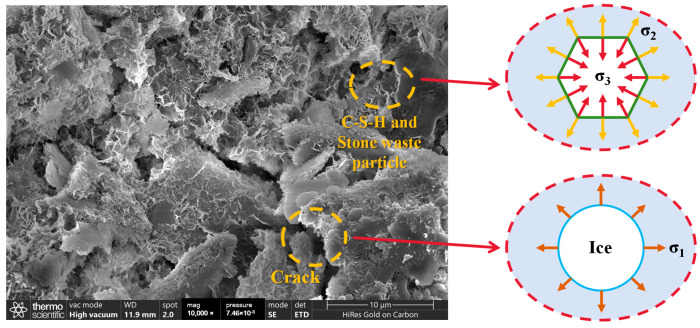
Schematic diagram of freeze–thaw cycle mechanism.

The inside of the specimen consists of substances such as free water, C–S–H, and stone waste particles, which have different physical properties. The freeze–thaw cycle consists of two phases: freezing and dissolution. In the freezing phase, the free water freezes and expands in volume, generating an ice expansion stress σ1 on the material around the pores. In addition, the C–S–H and the stone waste particles have different physical properties, which generate different contraction stresses in the freezing phase, σ2 for the C–S–H and σ3 for the stone waste particles (Figure 22).

All three forms of stresses lead to different degrees of cracks and holes inside the specimen. During the dissolution phase, all three stresses are gradually released as the temperature increases. Free water can enter the formed cracks and holes, and as the number of freeze–thaw cycles increases, more cracks and holes will appear and the already damaged parts will be further deteriorated.

Through macro and micro tests, it was determined that the strength deterioration that occurs in the stone waste road base materials after freeze–thaw action is mainly due to two reasons. Firstly, as the temperature continues to decrease, the free and capillary water inside the stone waste road base material gradually develops from the liquid state to the solid state. During the freezing process, the water in the specimen continuously freezes, leading to a slowdown in the rate of hydration reaction, which hinders the strength growth. Secondly, the volume of water increases after freezing, causing the displacement of the already formed gelling particles and stone waste particles, which destroys the cementation between the stone waste particles and the hydration products, and damages the already formed reticular skeleton structure of the hydration products, leading to the reduction in strength.

## 4. Conclusions

In this paper, the macroscopic and microscopic scale effects of the strength decay of stone waste road base materials were investigated by conducting dry–wet cycling tests and freeze–thaw cycling tests on two types of stone waste base materials, as well as by conducting unconfined compressive strength tests, SEM tests, and MIP tests on the specimens after cycling. The key results are summarized as follows:

(1) Through the apparent state of the specimen, it can be observed that the stone waste base material under the conditions of a dry–wet cycle and freeze–thaw cycle has undergone repeated uplift and contraction and the mutual dissolution of pore water, resulting in the damage phenomena of the coarsening of pore space, increasing of fissure, and hydrolysis of cement, which led to the decrease in the quality of the specimen and the decrease in the macroscopic strength. And, with the increase in the number of cycles, there is no obvious crack on the surface of the CGF waste stone road base material specimen, and the surface damage area of the cement stone waste base material specimen gradually increases.
(2) Under the condition of six times of dry–wet cycles, the unconfined compressive strength of the stone waste base material changes greatly, and with the increase in the number of cycles, the rate of decrease in the unconfined compressive strength of the specimen gradually decreases.
(3) The CGF stone waste road base material and cement stone waste road base material damage deterioration degree increase are larger at the beginning of the dry–wet cycle, and with the increase in the number of dry–wet cycle, the growth rate is gradually reduced. The CGF stone waste road base material’s resistance to dry–wet cycle ability is better than the cement stone waste road base material.
(4) After the MIP test pressure reaches 4.01 kPa, mercury rapidly enters into the pores of the stone waste road base material, and the CGF is the main peak of the stone waste road base material which gradually moves to the right with the increase in the number of dry–wet and freeze–thaw cycles, indicating that the stone waste road base material is more prone to deterioration reactions during freeze–thaw cycles. The slope of the fitted curve (*lnV–lnS*) increases with the number of dry–wet and freeze–thaw cycles, which suggests that the development of porosity is a function of the number of cycles for the practical application of stone wastes.
(5) The results of the SEM tests showed that after six dry–wet cycles, the CGF stone waste road base material and the cement stone waste road base material showed internal cracking and similar deterioration, with the degree of deterioration increasing with the number of dry–wet cycles and the degree of cracking intensifying, which is consistent with the strength results and the results of the MIP tests.

The analysis and discussion of the results of the experiments contribute to the subsequent research, which provides a direction and reference for further research.

## Figures and Tables

**Table 1:** Basic physical properties of stone waste.

Particle Density (GS)	Plastic Limit (%)	Fine Stone Waste (<0.005 mm)			
		Liquid Limit (%)	Plastic Limit (%)	Plasticity Index (Ip)	Liquidity Index (IL)
2.68	15.6	39.5	18.9	20.6	1.1

**Table 2:** Durability research program.

Specimen Number	Type of Binder	Binder Content (%)	Water–Binder Ratio	Curing Time (Days)	Tests	Number of Test Groups
C5	CGF	5%	1	28	Dry–wet cycle Freeze–thaw cycle UCST MIP SEM–EDS	3
C10	CGF	10%				
C15	CGF	15%				
C20	CGF	20%				
S5	Cement	5%				
S10	Cement	10%				
S15	Cement	15%				
S20	Cement	20%				

**Table 3:** Test apparatus and methods.

Test	Test Instrument Parameters	Test Norms
Dry–wet cycle	Constant temperature and humidity maintenance box, YH-40B Electrothermal constant temperature blast drying box, DHG-9245A	JTG E51-2009 [32]
Freeze–thaw cycle	Freezing and thawing box, ZT-CTH-225L	JTG E51-2009 [32]
UCST	Strain-controlled and the rate of vertical displacement was fixed at 1%/min.	GB/T50123-2019 [33]
SEM	HITACHI S-4800, secondary electron imaging resolution: 1.0 nm@15 KV, magnification: 20×–800,000×, accelerating voltage: 0.1–30 KV, sample stage: three-axis motor stage, and maximum sample size: Φ100 mm.	JBT 6842-1993 [35]
MIP	Micro Active AutoPore V 9600.	GB/T 21650.1-2008 [36]

## Data Availability

The data presented in this study are available upon request from the corresponding author. The data are not publicly available due to the confidentiality of the subject research.

## References

[1] Dimitriou G., Savva P., Petrou M.F. (2018). Enhancing mechanical and durability properties of recycled aggregate concrete. Constr. Build. Mater..

[2] Torres A., Brandt J., Lear K., Liu J. (2017). A looming tragedy of the sand commons. Science.

[3] Entwisle D.C., Hobbs P.R.N., Jones L.D., Gunn D., Raines M.G. (2005). The Relationships between Effective Porosity, Uniaxial Compressive Strength and Sonic Velocity of Intact Borrowdale Volcanic Group Core Samples from Sellafield. Geotech. Geol. Eng..

[4] Thakur A.K., Pappu A., Thakur V.K. (2018). Resource efficiency impact on marble waste recycling towards sustainable green construction materials. Curr. Opin. Green Sustain. Chem..

[5] Prakash B., Saravanan T.J., Kabeer K.I.S.A., Bisht K. (2023). Exploring the potential of waste marble powder as a sustainable substitute to cement in cement-based composites: A review. Constr. Build. Mater..

[6] Li H., Huang F., Cheng G., Xie Y., Tan Y., Li L., Yi Z. (2016). Effect of granite dust on mechanical and some durability properties of manufactured sand concrete. Constr. Build. Mater..

[7] Yang R., Yu R., Shui Z., Gao X., Han J., Lin G., Qian D., Liu Z., He Y. (2020). Environmental and economical friendly ultra-high performance-concrete incorporating appropriate quarry-stone powders. J. Clean. Prod..

[8] Wu Y.L., Yang J.J. (2022). Soil Stabilizer–Materials·Mechanism Applications.

[9] Arel H.Ş. (2016). Recyclability of waste marble in concrete production. J. Clean. Prod..

[10] Joudi-Bahri I., Lecomte A., Ouezdou M.B., Achour T. (2012). Use of limestone sands and fillers in concrete without superplasticizer. Cem. Concr. Compos..

[11] Ren Q., Xie M., Zhu X., Zhang Y., Jiang Z. (2020). Role of limestone powder in early-age cement paste considering fineness effects. J. Mater. Civ. Eng..

[12] Munir M.J., Kazmi S.M.S., Wu Y. (2017). Efficiency of waste marble powder in controlling alkali–silica reaction of concrete: A sustainable approach. Constr. Build. Mater..

[13] Kirgiz M.S. (2015). Use of ultrafine marble and brick particles as raw materials in cement manufacturing. Mater. Struct..

[14] Wu Y.L., Yang J.J., Chang R.Q. (2023). The design of ternary all-solid-waste binder for solidified soil and the mechanical properties, mechanism and environmental benefits of CGF solidified soil. J. Clean. Prod..

[15] Wang F., Meng F., Feng T., Wang Y., Jiang J., Shi J. (2023). Effect of stone powder content on the mechanical properties and microstructure of tunnel slag aggregate-based concrete. Constr. Build. Mater..

[16] Zheng S., Liang J., Hu Y., Wei D., Lan Y., Du H., Rong H. (2021). An experimental study on compressive properties of concrete with manufactured sand using different stone powder content. Ferroelectrics.

[17] Campos H.F., Klein N.S., Marques Filho J., Bianchini M. (2020). Low-cement high-strength concrete with partial replacement of Portland cement with stone powder and silica fume designed by particle packing optimization. J. Clean. Prod..

[18] Chen R., Jiao Y., Xiao M., Yang H., Wang C. (2024). Effect of Composition Characteristics on Mechanical Properties of UHPMC Based on Response Surface Methodology and Acoustic Emission Monitoring. Materials.

[19] Shen W., Wu J., Du X., Li Z., Wu D., Sun J., Wang Z., Huo X., Zhao D. (2022). Cleaner production of high-quality manufactured sand and ecological utilization of recycled stone powder in concrete. J. Clean. Prod..

[20] Bakis A. (2019). Increasing the Durability and Freeze-Thaw Strength of Concrete Paving Stones Produced from Ahlat Stone Powder and Marble Powder by Special Curing Method. Adv. Mater. Sci. Eng..

[21] Chen J. (2021). Experimental analysis of influence factors on pavement concrete performance: Different content of stone powder in manufactured sand. Mater. Des. Process. Commun..

[22] Ministry of Ecology and Environment of the People’s Republic of China (2022). Annual Report on Prevention and Control of Solid Waste Pollution in Large and Medium-sized Cities of China in 2022. https://www.mee.gov.cn/hjzl/sthjzk/sthjtjnb/202312/t20231229_1060181.shtml.

[23] Ministry of Ecology and Environment, People’s Republic of China (2023). Annual Report on China’s Ecological and Environmental Statistics 2021. https://www.mee.gov.cn/hjzl/sthjzk/sthjtjnb/202301/t20230118_1013682.shtml.

[24] Wu Y.L., Yang J.J., Chang R.Q., Li S., Kou H. (2024). Strength, leaching characteristics and microstructure of CGF+P all-solid-waste binder solidification/stabilization Cu(II) contaminated soil. Constr. Build. Mater..

[25] Qiu X.Y., Yang J.J., Wu Y.L., Yan L., Liu Q. (2024). Effect of fiber content on mechanical properties of fiber-reinforced CGF All-Solid-Waste binder-solidified soil. Materials.

[26] Liu Q., Yang J.J., Wu Y.L., Wang Z.M., Qiu X., Yan L. (2024). Physical and mechanical properties of All-Solid-Waste-Based binder-modified abandoned marine soft soil. J. Mar. Sci. Eng..

[27] Gi W., Monamed A., Khalid N.H., Nor H.M., Hainin M.R., Jaya R.P., Sani W.N.H.M., Ismail C.R., Aziz M.M.A. (2019). Recycled concrete aggregate as a road base material. Mater. Sci. Eng..

[28] Koleva D.A., Breugel K.V., Wit J.H. (2008). Correlation of microstructure, electrical properties and electrochemical phenomena in reinforced mortar. Breakdown to multiphase interface structures. Part I: Microstructural observations and electrical properties. Mater. Charact..

[29] Care S. (2008). Effect of temperature on porosity and on chloride diffusion in cement pastes. Constr. Build. Mater..

[30] Wang M., Yang J.J., Wu Y.L., Lu Y. (2024). Rapid predictive method for the deterioration depth of cement solidified marine soft soil. Soils Found..

[31] Wang M., Yang J., Wu Y., Yan L. (2024). Laboratory Test study on deterioration process of cement soil piles in radial direction in k0 consolidated marine soft soil foundation. Measurement.

[32] (2009). Test Methods of Materials Stabilized with Inorganic Binders for Highway Engineering.

[33] (2019). Standard for Geotechnical Testing Method.

[34] Jiao Z., Wang Y., Zheng W., Huang W. (2018). Effect of dosage of sodium carbonate on the strength and drying shrinkage of sodium hydroxide based alkali-activated slag paste. Constr. Build. Mater..

[35] (1993). Scanning Electron Microscope Test Methods.

[36] (2008). Determination of Pore Size Distribution and Porosity of Solid Materials by Mercuric Pressure and Gas Adsorption Methods, Part 1.

[37] Kim J.H., Choi S.W., Lee K.M., Choi Y.C. (2018). Influence of internal curing on the pore size distribution of high strength concrete. Constr. Build. Mater..

[38] Huang J., Zhang Y., Sun Y., Ren J., Zhao Z., Zhang J. (2021). Evaluation of pore size distribution and permeability reduction behavior in pervious concrete. Constr. Build. Mater..

